# Professional Pecksniffs

**Published:** 1860-07

**Authors:** 


### ARTICLE XVIII.

### *Professional Pecksniffs.*

Censorius physicians have sometimes complained of pious brethren, disposed to make their religious connections useful in a business way, who obtrude their sanctimoniousness on the profession, relating their experience before medical societies, or enriching the journals with descriptions of the illness of Elder Sniffle's child, or the miraculous recovery of the Rev. Mr. Honeyman's grandmother. An amusing specimen of this class of practitioners, among whom the affectation of godliness supplies the want of knowledge and skill, is found in Mr. Sutleffe, of London, from whose work* we are about to make a few extracts:

*Hoarhound Tea keeps a saint out of Heaven upwards of 24 years*—"In the summer of 1800, I was asked if I wished to see a triumphant saint expire. 'Much more,' I replied, 'than to see Rome in all her pristine or present glory.' I was accordingly directed to call on Mrs. W——, of the Surrey Road, which I did, in whom I beheld the nearest approach to an animated skeleton I ever expect to see. She instantly recognized me (having often met me at the sanctuary,) and shook hands feebly. She was on the mount of God's unchanging love."

Mr. Sutleffe had the cruelty to administer hoarhound tea, which prevented the saint from "going home."

* *Medical and Surgical Cases; selected during a practice of* 38 *years.* By Edward Sutleffe. 8vo. pp. 628. London, 1824. The book is now rare.

When informed she was out of danger, she shed tears of grief. She shortly afterwards retired to Warwickshire instead of Paradise, and grew quite lusty.

*Fatal Hemorrhage from a Bubo.*—Mr. Sutleffe was called to a young man with a sloughing bubo, where "the pulsation from the iliac artery was awful." The artery burst, and the patient quickly bled to death. "I have since thought that a ligature applied to the artery might have arrested the bleeding, if not have saved his life." On returning home, Dr. Sutleffe reflected on these words of the Apocrypha: "Oh, Adam! what hast thou done?" We presume our readers will add: Oh, Sutleffe, what didst thou *leave undone*.

*Aphonia.*—Our author, in common with his tribe, is an admirer of old women, and observes, "that old women do as well as old men, and sometimes better." In illustration of this aphorism, he mentions two cases of aphonia which baffled him, but were speedily cured by an old woman with "gin and oatmeal."

*Flannel.*—Dr. Sutleffe expresses a mortal antipathy to the wearing of flannel, quoting the prohibition of the prophet Ezekiel (chap. xliv,) "They shall not gird themselves with wool that causeth sweat."

*Uric Acid.*—A compound of rhubarb, soap, and juniper is recommended by Mr. Sutleffe, "to sweep out the kidneys, ureters, and bladder, incommoded with red gravel. I call it my *besom*, and it is thought to be an appropriate name in the circle in which I move."

*Mania.*—Mr. Sutleffe advocates the use of ground ivy in insanity. "I cannot call to mind a single case of mania where the *glecoma hederacea* has had a full trial, without eventual recovery."

*Puerperal Fever.*—A "scripturally pious" lady had puerperal fever, which completely baffled Mr. Sutleffe and his friend Dr. Sims. They had the mortification to witness "deeper advances toward despondency," in the midst of which "the scene partook not of the house of mourning, but was rather an epitome of the abode of bliss." Being pressed for a prognosis, our author stated: "that in reference to the laws of the obstetric art, the patient must die; but as Dr. Sims and himself had now turned over the case to God, it was possible that she might recover." It does not appear that the DEITY took charge of this case, for the lady died. "Fraught with instruction in its progressive stages, this case was singularly honored in its termination; for the children of the deceased came forth with one consent to join themselves in a perpetual covenant to the Lord and his church, where they now shine as polished pillars in the temple of grace."

[*Cincinnati Lancet and Observer.*

